# PECAM-1 Is Down-Regulated in γδT Cells during Remission, but Up-Regulated in Relapse of Multiple Sclerosis

**DOI:** 10.3390/jcm11113210

**Published:** 2022-06-04

**Authors:** Michał K. Zarobkiewicz, Izabela Morawska, Wioleta Kowalska, Paweł Halczuk, Jacek Roliński, Agnieszka A. Bojarska-Junak

**Affiliations:** 1 Department of Clinical Immunology, Medical University of Lublin, 20-093 Lublin, Poland; 51977@student.umlub.pl (I.M.); wioleta.kowalska@umlub.pl (W.K.); jacek.rolinski@umlub.pl (J.R.); 2 Department of Neurology, Medical University of Lublin, 20-090 Lublin, Poland; pawel.halczuk@umlub.pl; 3 Department of Histology and Embryology with Experimental Cytology Unit, Medical University of Lublin, 20-080 Lublin, Poland

**Keywords:** PECAM-1, NKRP1A, γδ T, MS, multiple sclerosis

## Abstract

**Introduction**. PECAM-1 and NKRP1A are both involved in the vascular transmigration of T lymphocytes. Vascular transmigration is a crucial process in multiple sclerosis pathogenesis. **Methods and aim**. The current paper presents an analysis of PECAM-1 and NKRP1A expression on γδ T cells. Expression of PECAM-1 and NKRP1A on subsets of γδ T cells was performed with flow cytometry. **Results**. Based on the flow cytometry data, PECAM1 was slightly differentially modulated on γδ T cells—it was up-regulated during relapse, but down-regulated during remission. Moreover, a significant downregulation of CD3 expression was noted on γδ T cells from MS patients, most notably during relapse. **Conclusions**. This may be a sign of the overall activation of γδ T cells in the course of multiple sclerosis.

## 1. Introduction

Multiple sclerosis (MS) is a chronic, demyelinating disease of the central nervous system (CNS) with autoimmune background and increasing incidence and prevalence during recent years, especially in women [1,2,3]. MS is considered the most commonly diagnosed inflammatory neurological disorder in the young adult population [4]. MS leads not only to disability but also mental health problems, job loss or other socioeconomic difficulties [5,6,7]. Despite advances in the understanding of MS pathogenesis, there are still numerous gaps in our knowledge and consequently, the causal treatment is still unknown [8].

Natural killer cell surface protein P1A (NKRP1A/CD161) is a family of receptors with both activating and inhibitory properties, expressed mostly on natural killer (NK) cells as well as on T lymphocytes and dendritic cells (DC) [9,10]. In mice, the following receptors within the NKRP1A family can be distinguished: NK-RP1A, NKp80, and NKp65, while in humans the only receptor is NKRP1A [11]. The functions of NKRP1A receptor depends on the cell type it is expressed on. In the case of NK cells it shows inhibitory properties, and on T cells acts as activating receptor, stimulating the production of pro-inflammatory cytokines such as IL-17 and IFN-γ [12]. NKRP1A has been shown to be expressed on γδ T cells as well [13,14].

Platelet endothelial cell adhesion molecule (PECAM-1/CD31) is a protein present on a variety of cells within the vascular compartment, including different subpopulations of lymphocytes [15]. The role of PECAM-1 during inflammation is diverse and wide, as they are involved in many mechanisms such as transmigration of leukocytes through intracellular junctions, apoptosis, angiogenesis, and many others [16,17,18]. In several pathways, PECAM-1 serves as a signaling molecule [17]. In patients with multiple sclerosis, there is an increased expression of different cell adhesion molecules in the central nervous system, especially around the lesions, in cerebral microvessels, and on local immune cells [19,20,21]. Furthermore, expression of PECAM-1 in patients with MS is upregulated in serum, plasma and cerebrospinal fluid [22,23,24]. Moreover, PECAM-1 might be involved in stabilizing the blood–brain barrier, the integrity of which is crucial for preventing neuroinflammation [24,25]. γδ T cells, particularly the Vδ1 subset, also express PECAM-1 protein [26].

γδ T cells form a small subset of T cells, comprising usually up to 5% thereof; human γδ T can then be further divided into three major subsets based on δ chain expression, namely Vδ1, Vδ2 and Vδ3 [27]. Due to the lack of specific anti-Vδ3 antibodies, that subset is relatively poorly understood. Vδ1 and Vδ2 cells differ in some aspects—the former recognises non-classical MHC molecules, e.g., MIC-A, MIC-B and ULBPs, while the latter responds to phosphoantigens that accumulate in neoplastic cells and as a result of some bacterial infections [27]. Moreover, Vδ1 tends to preferentially express PECAM-1, while Vδ2 seems to be more skewed towards NKRP1A [26]. Both receptors are important for transmigration through vascular endothelium [28].

In our recent study, we have observed a significant upregulation of RORγT, a classical Th17-related transcription factor, in iNKT and γδ T cells during relapse in relapsing-remitting multiple sclerosis [29]. The aim of the current study is to further evaluate Th17-like γδ T and total γδ T cells in MS with a focus on the expression of two adhesion molecules—PECAM-1 and NKRP1A.

## 2. Materials and Methods

### 2.1. Study Group

The study group consisted of 29 patients diagnosed with the relapsing-remitting form of multiple sclerosis, recruited to the study at the Department of Neurology, the Medical University of Lublin after meeting the appropriate inclusion criteria. Inclusion criteria for the study group were described as: no other autoimmune diseases diagnosed, no glucocorticoid intake in the last 4 weeks before blood donation, no medical history of neoplasms, no history of neurosurgery procedures. In the control group, those criteria were described as: no hospitalization in the last 6 weeks before blood sampling, no autoimmune diseases, neurological diseases and neoplasms diagnosed, no diagnosed MS in first-degree relatives, no neurosurgical procedures in the medical history. Twenty-three patients were in remission and six during the relapse; eighteen of the patients were treated with natalizumab, eight patients with cladribine, fingolimod or immu-838 and three patients were not treated. The control group consisted of 15 healthy volunteers with similar age and sex distribution. More information is provided in Table 1. Blood samples were taken from patients and healthy volunteers after they had signed written informed consent. Blood was taken into EDTA-coated tubes and processed immediately. The study protocol was approved by the Bioethical Committee at the Medical University of Lublin.

### 2.2. Flow Cytometry

**Figure 1:**
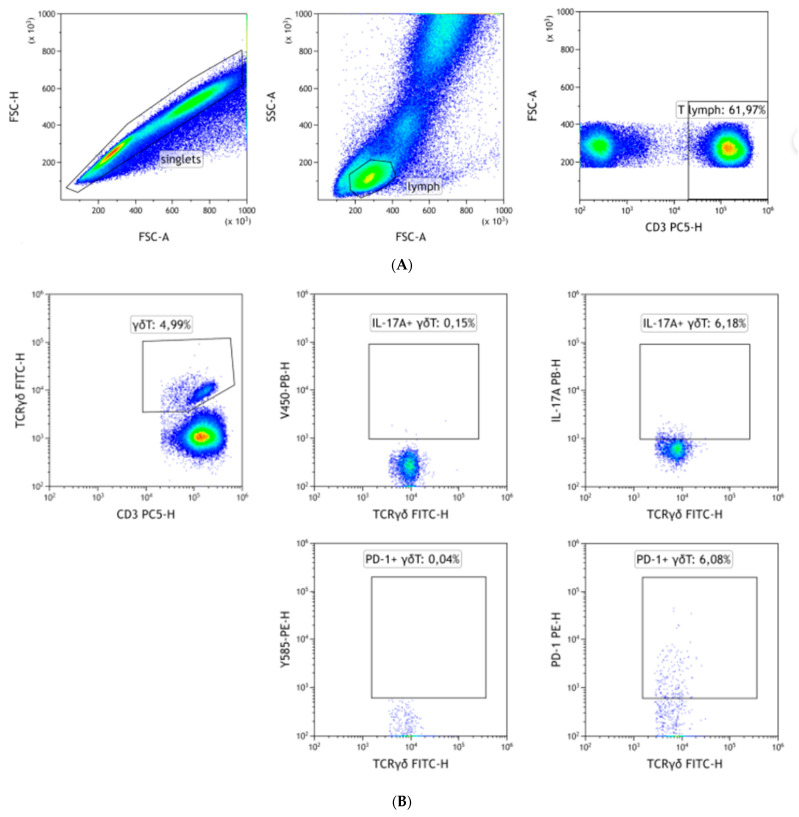

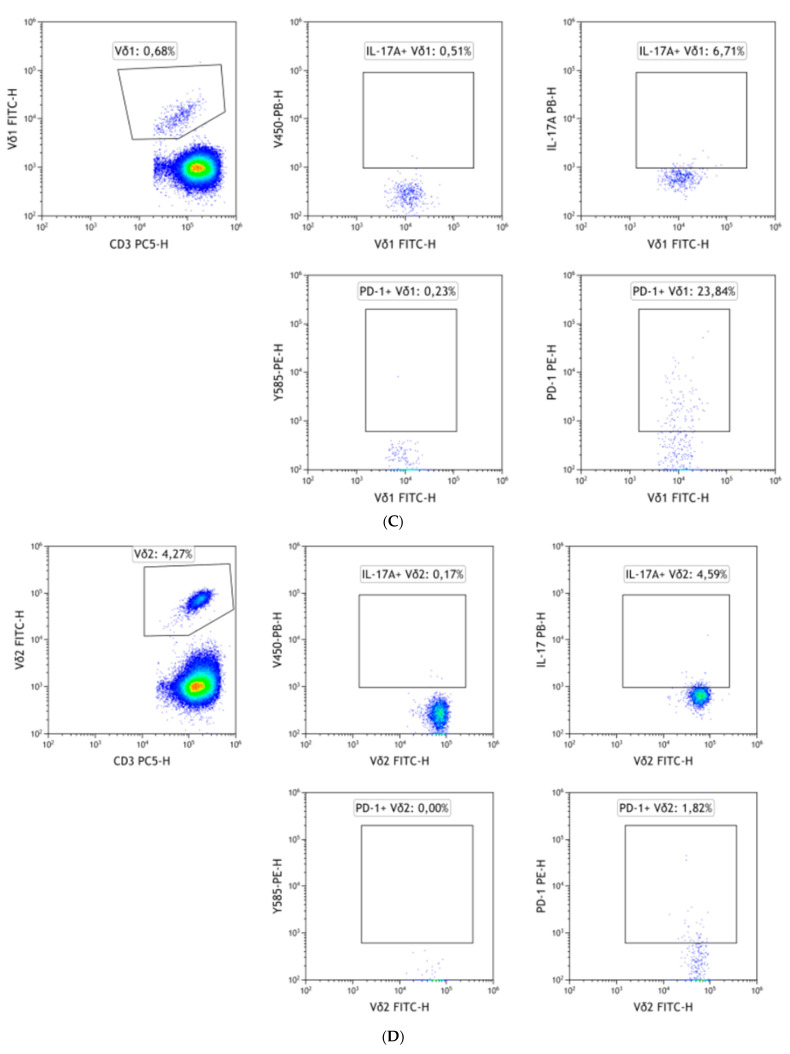
Gating strategy. Initially, singlets were gated based on FSC-H/FSC-A. Next, a lymphocyte gate was applied on SSC-A/FSC-A. Finally, T lymphocytes were gated as CD3+ cells (**A**). Then, γδ T, Vδ1 or Vδ2 cells were gated among CD3+ cells. Expression of IL-17A and PD-1 was assessed as a percentage of positive cells, thus FMO (**left**) controls were used for gating (**B**–**D**). PECAM-1 and NKRP1A expression was quantified using MFI instead.

100 uL of peripheral blood was stained with anti-human antibodies against surface antigens: FITC anti-human TCRγδ (BioLegend, San Diego, CA, USA; #331208), FITC anti-human Vδ1 (ThermoFisher, Waltham, MA, USA; #TCR2730), FITC anti-human Vδ2 (BioLegend, #331406), PE-Cy5 anti-human CD3 (BioLegend, #344866), Alexa Fluor 700 anti-human CD161 (BioLegend, #339942) and APC-Cy7 anti-human CD31 (BioLegend, #303120), PE anti-human PD-1 (BD, Franklin Lakes, NJ, USA; #557946). Blood was incubated at room temperature in the dark for 20 min. BD FACS Lysing Solution was then added to each tube and incubated for 10 min at room temperature in the darkness, thus cells were permeabilized, fixed and erythrocytes were lysed [30,31]. Samples were then centrifuged and washed twice with PBS. In the next step, antibodies against intracellular antigens (Pacific Blue anti-human IL-17A; BioLegend, #512312) were added and incubated for 20 min in the dark at room temperature. Once again samples were washed with PBS. CytoFlex LX (Beckmann Coulter, Brea, CA, USA) was used for sample acquisition. The gating strategy is summarised in Figure 1. The detailed configuration of cytometers used in the study is presented in Supplementary Table S1. Kaluza (Beckmann Coulter) was used to analyse flow cytometry data. Data are either presented as percentage of positive cells or as mean fluorescence intensity (MFI). MFI is a measure of the density of antigen expression, it is especially useful for antigens where no clear cut-off value between positive and negative cells can be established.

### 2.3. Cell Sorting

PBMCs were isolated using Gradisol L (Aqua-Med, Łódź, Poland) in a density gradient. Isolated cells were stained with anti-human monoclonal antibodies PE-Cy7 anti-human CD3 (BD, #563423) and FITC anti-human TCRγδ (BioLegend, #331208), incubated for 20 min at room temperature in the dark, and then washed with 2 mL of PBS. γδ T were then sorted with BD FACS Aria IIu (Becton Dickinson, Franklin Lakes, NJ, USA). Each time after sorting, the purity of cells was assessed, samples were further processed only if the purity was >95%. Cells were suspended in the RLT buffer (Qiagen, Inc., Valencia, CA, USA) with β-mercaptoethanol and immediately frozen at −80 °C.

### 2.4. RT-qPCR

Total RNA was isolated with Blood RNA Mini Kit (Qiagen), the manufacturer’s manual was followed. Then, cDNA was synthesized with the Transcriptor First Strand cDNA Synthesis Kit (Roche Applied Science, Mannheim, Germany). cDNA was then used for qPCRs. TaqMan (ThermoFisher, Applied Biosystems, Austin, TX, USA) probes for CD31 (Hs00169777_m1) and CD161 (Hs00174469_m1) were used to quantify mRNA expression, Human GAPDH Endogenous Control (ThermFisher) was used to quantify home-keeping gene expression. Reactions were set up with The TaqMan FastAdvanced MasterMix (ThermoFisher) in 20 μL and ran with Applied Biosystems 7300 Real-Time PCR System (ThermoFisher). The relative expression was calculated as 2^−ΔCT^. ΔCT is calculated as (Ct gene of interest—Ct internal control).

### 2.5. Statistical Analysis

Data were analysed with GraphPad Prism 8 (GraphPad Software, San Diego, CA, USA). The Shapiro–Wilk test was used to assess data distribution. The Kruskal–Wallis test with Dunn–Šidák correction was employed for flow cytometry data analysis while U Mann–Whitney for qPCR data. The data are presented as the median and interquartile range (IQR). Statistical correlations were calculated with the Spearman test.

## 3. Results

### 3.1. CD3 Is Significantly Down-Regulated on γδ T Cells

**Figure 2:**
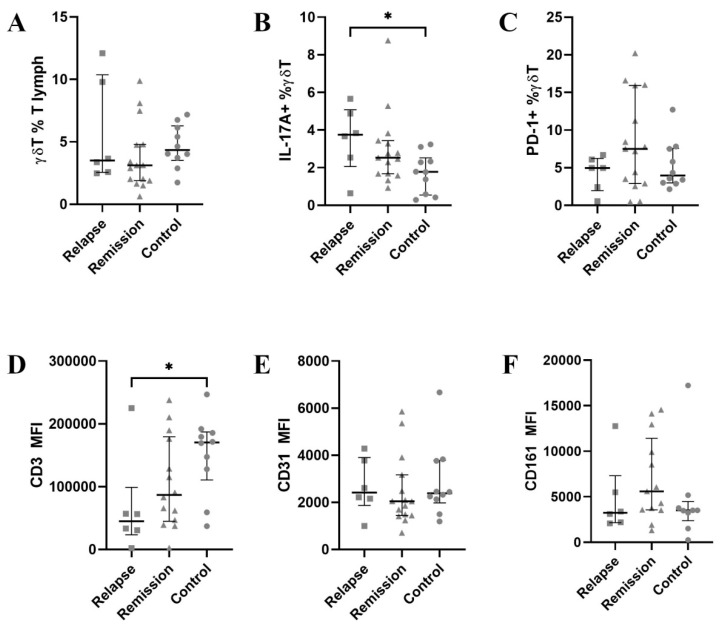
The results for the whole γδ T population. A horizontal line with whiskers represents a median with an inter-quartile range (IQR). (**A**)—percentage of γδ T among total T cells, (**B**)—percentage of IL-17+ γδ T cells, (**C**)—percentage of PD-1+ γδ T cells, (**D**–**F**)—MFI values for CD3, CD31 and CD161 respectively. MFI—mean fluorescence intensity. * denotes *p* < 0.05.

At first, we analysed the percentage of γδ T cells in peripheral blood along with IL-17A and the expression of PD-1 on them. γδ T cells were insignificantly decreased in MS patients (Figure 2A). IL-17A expression was significantly higher in relapse than in the control group (Figure 2B, *p* < 0.05). Contrary, PD-1 expression was the highest during remission (Figure 2C, *p* = 0.34). We have also noticed a different expression of CD3, thus we have then compared CD3 MFI (mean fluorescent intensity) values—indeed, MS patients, especially in relapse, had around the 2-fold lower expression of CD3 (Figure 2D). The expression of NKRP1A and PECAM-1 had similar expression levels in all groups (Figure 2E,F).

### 3.2. PECAM-1 Is Slightly Down-Regulated in Vδ1 during Remission

**Figure 3:**
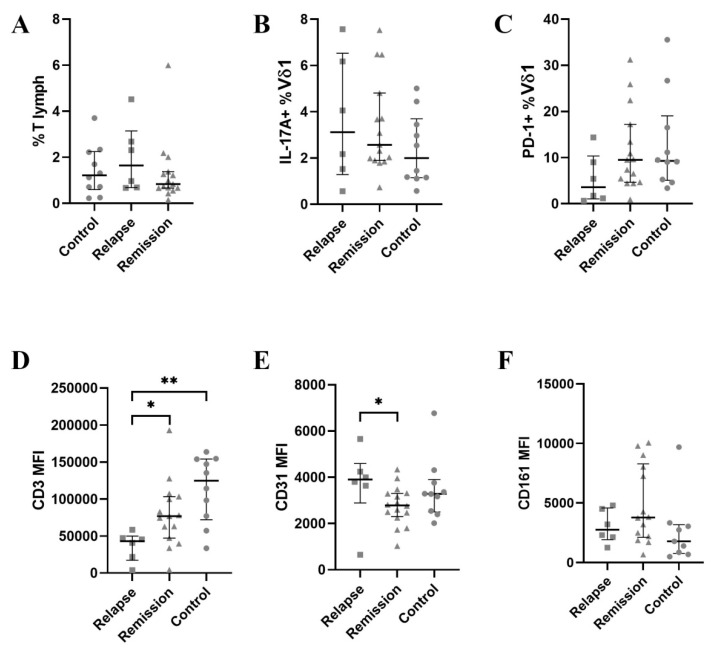
The results for the Vδ1 subset. The horizontal line with whiskers represents the median with inter-quartile range (IQR). (**A**)—percentage of Vδ1 among total T cells, (**B**)—percentage of IL-17+ Vδ1 cells, (**C**)—percentage of PD-1+ Vδ1 cells, (**D**–**F**)—MFI values for CD3, CD31 and CD161 respectively. MFI—mean fluorescence intensity. * denotes *p* < 0.05, ** *p* < 0.01.

Then, we analysed a Vδ1 subset. The percentage of Vδ1 was insignificantly higher in MS patients than in controls (Figure 3A). Similarly to total γδ T cells, PD-1 expression was lower in relapse (Figure 3C). Moreover, CD3 expression was almost three-fold lower in relapse than in the control group (Figure 3D). Finally, PECAM-1 was insignificantly down-regulated in remission compared to the relapse and control group, while NKRP1A showed a very slight up-regulation in remission (Figure 3E).

### 3.3. CD3 Is Further Down-Regulated in Vδ2 Cells

**Figure 4:**
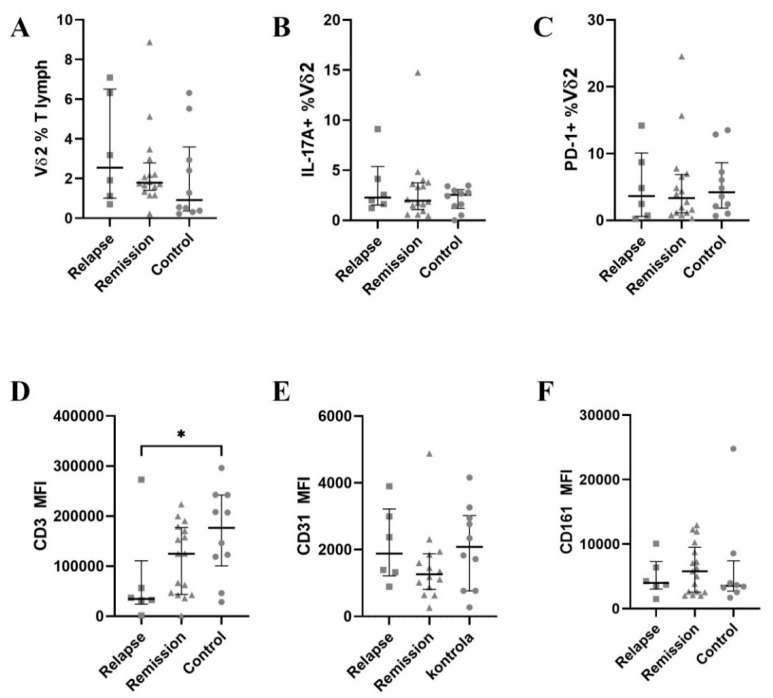
The results for the Vδ2 subset. A horizontal line with whiskers represents a median with inter-quartile range (IQR). (**A**)—percentage of Vδ2 among total T cells, (**B**)—percentage of IL-17+ Vδ2 cells, (**C**)—percentage of PD-1+ Vδ2 cells, (**D**–**F**)—MFI values for CD3, CD31 and CD161 respectively. MFI—mean fluorescence intensity. * denotes *p* < 0.05.

The percentage of Vδ2 was insignificantly higher in MS patients, especially during relapse (Figure 4A). PECAM-1 expression on Vδ2 was similar during relapse and in control subjects, but was lower in remission, while NKRP1A was insignificantly up-regulated in remission (Figure 4E). The most striking difference was observed for CD3 expression, which was more than 2-fold lower in relapse than in control (Figure 4D).

### 3.4. PECAM-1 mRNA Is Up-Regulated While NKRP1A mRNA Is Downregulated in γδ T during Relapse

**Figure 5:**
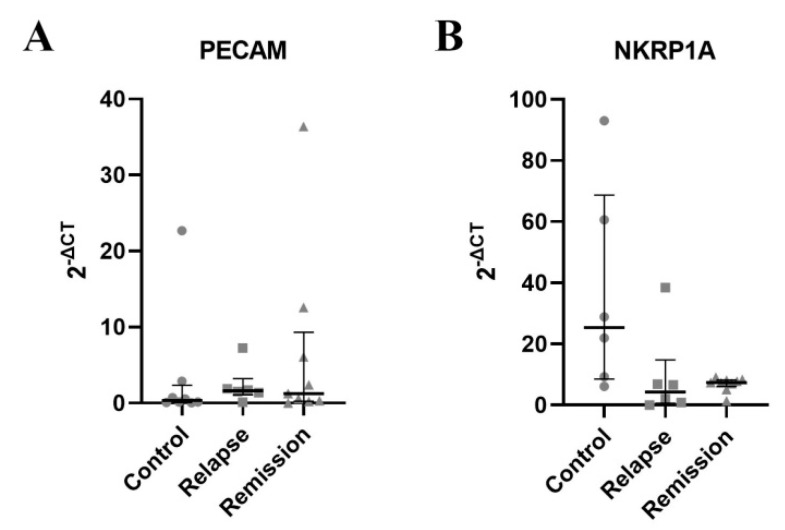
The results for the NKRP1A and PECAM-1 mRNA expression in sorted γδ T cells. A horizontal line with whiskers represents the median with inter-quartile range (IQR). (**A**)—expression of PECAM1 mRNA, (**B**)—expression of NKRP1A mRNA.

Finally, we further confirmed our results in RT-qPCR. mRNA expression for PECAM-1 tended to be higher in relapse (Figure 5A). Moreover, a noticeable drop in mRNA expression of NKRP1A was observed during relapse (Figure 5B).

### 3.5. Correlations

Correlations between clinical (annual relapse rate [ARR], expanded disability status scale [EDSS], years from diagnosis, age) and immunological factors (percentages of γδ T, Vδ1, Vδ2, γδ T dim, γδ T bright, expression of CD31, CD161 and CD3 on γδ T, Vδ1 and Vδ2) were calculated with Spearman test. Both the expression of CD3 and CD31 on Vδ1 cells significantly correlated with ARR with r = 0.61 and r = −0.65 respectively. The expression of CD31 and CD161 on all three subsets strongly positively correlated between the subsets. The full correlation matrix can be found in Supplementary Table S2.

## 4. Discussion

The most important observation from the current study is a significant decrease in CD3 expression in γδ T cells from MS patients. The decrease in CD3 expression may potentially be a result of TCR-dependent activation of γδ T lymphocytes [32,33]. A decrease in surface CD3ε was also noted in cancer patients—both in tumour-infiltrating lymphocytes and peripheral blood T lymphocytes—in-vitro studies suggest that this may possibly be mediated by tumour-derived microvesicles [34]. Moreover, in conventional T cells, CD3 is naturally expressed at different levels in various subsets, e.g., FoxP3+ cells have significantly higher surface expression of CD3 than FoxP3- ones [35].

PECAM-1 is a molecule that serves a plethora of different functions. PECAM-1 seems to control the activation of T cells in order to prevent AICD (activation-induced cell death) due to overstimulation [36]. PECAM-1 cytoplasmic domain with ITIM motifs is phosphorylated after TCR-mediated activation, protein-tyrosine phosphatases are then recruited and eventually, TCR signalling is inhibited [37]. Moreover, PECAM-1 is known to be preferentially expressed by naive Th cells [38]. In several autoimmune murine models, including experimental autoimmune encephalomyelitis, PECAM-1-deficiency leads to a more severe disease course [37]. Furthermore, an increased level of soluble PECAM-1 was observed among MS patients [23,24]. PECAM-1 apart from lymphocytes is also expressed on vascular endothelial cells and is most probably important for the maintenance of the blood–brain barrier [25]. Nevertheless, we have not observed any significant difference in PECAM-1 expression between MS patients and healthy controls neither in the case of pan-γδ T cells nor for Vδ1 and Vδ2 subsets.

Interestingly, our results are mostly in-line with Poggi et al. who observed a distinct expression pattern of NKRP1A and PECAM-1 on γδ T cells [28]. They observed a high PECAM-1 and low NKRP1A expression on Vδ1 and the opposite on Vδ2 cells. Indeed, functional studies proved that Vδ1 preferentially use PECAM-1 for endothelial transmigration, while Vδ2 tends to use NKRP1A [39]. Indeed, previous studies show that NKRP1A is an important molecule for Vδ2 transmigration in both healthy volunteers and MS patients [26]. Moreover, NKRP1A+ γδ T cells are significantly up-regulated in the cerebrospinal fluid of MS patients [40]. Similar overexpression of NKRP1A+ cytotoxic T cells was previously observed in MS patients [41].

**Figure 6:**
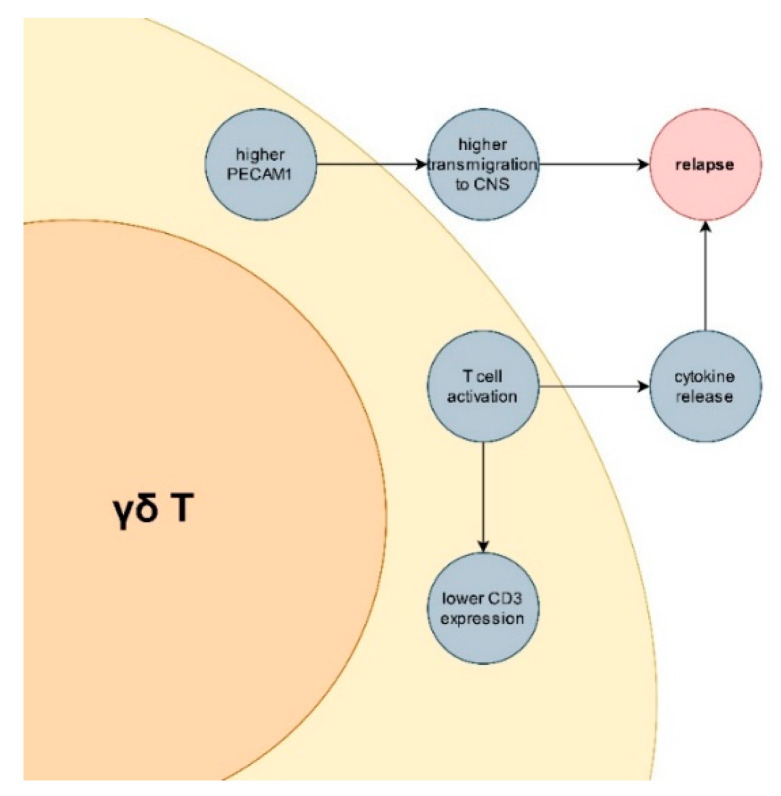
Summary of major findings and their theoretical implications for multiple sclerosis immunopathogenesis.

Lymphocyte transmigration through the blood–brain barrier constitutes an important step in MS pathogenesis [42]. The importance of the blood–brain barrier has been recently thoroughly reviewed by Schreiner et al. [43]. Indeed, two modern and highly effective drugs (natalizumab and fingolimod) target the transmigration of lymphocytes [44,45]. Differences in PECAM1 and NKRP1A expression between MS patients (especially in relapse) and healthy controls seems to reflect increased transmigratory potential. Moreover, down-regulation of CD3 suggest wide-spread activation of γδ T cells in MS, especially during relapse. We have previously reported a higher potential for IL-17A production by γδ T lymphocytes in MS patients [29]. Bearing in mind the importance of IL-17A in MS pathogenesis [46,47], it seems that γδ T lymphocytes may play an important role both in immunopathogenesis of MS in general and also in pathogenesis of MS relapses. Major findings and their possible implications are summarized in Figure 6.

The current study has some important limitations. Most importantly, it was carried out on a limited sample size which consisted of patients without any treatment and those already treated. Moreover, no analysis of cerebrospinal fluid γδ T cells was performed.

## 5. Conclusions

We have observed a significant activation of γδ T cells in MS patients, especially pronounced during relapse. Along with previously reported results, this suggests a possible important role of γδ T cells in MS.

## Figures and Tables

**Table 1:** Basic information about study groups; EDSS—expanded disability status scale; ARR—annual relapse rate. Values are presented as median, IQR.

	% Women	Age	EDSS	ARR	Years after Diagnosis
MS patients	76.47%	43 (29.5–51)	3.5 (3–5.5)	0 (0–1)	10 (7.5–14.75)
Healthy volunteers	70.00%	44 (26–58)			

## Data Availability

The data presented in this study are available on request from the corresponding author.

## Supplementary Materials

The following supporting information can be downloaded at: https://www.mdpi.com/article/10.3390/jcm11113210/s1, Table S1: The detailed configuration of cytometers; Table S2: Spearman correlation matrix for major clinical and immunological parameters.

## References

[1] Sorensen P.S., Sellebjerg F., Hartung H.-P., Montalban X., Comi G., Tintoré M. (2020). The Apparently Milder Course of Multiple Sclerosis: Changes in the Diagnostic Criteria, Therapy and Natural History. Brain.

[2] Koch-Henriksen N., Sørensen P.S. (2010). The Changing Demographic Pattern of Multiple Sclerosis Epidemiology. Lancet Neurol..

[3] Koch-Henriksen N., Thygesen L.C., Stenager E., Laursen B., Magyari M. (2018). Incidence of MS Has Increased Markedly over Six Decades in Denmark Particularly with Late Onset and in Women. Neurology.

[4] Wallin M.T., Culpepper W.J., Nichols E., Bhutta Z.A., Gebrehiwot T.T., Hay S.I., Khalil I.A., Krohn K.J., Liang X., Naghavi M. (2019). Global, Regional, and National Burden of Multiple Sclerosis 1990–2016: A Systematic Analysis for the Global Burden of Disease Study 2016. Lancet Neurol..

[5] Davis B.E., Lakin L., Binns C.C., Currie K.M., Rensel M.R. (2021). Patient and Provider Insights into the Impact of Multiple Sclerosis on Mental Health: A Narrative Review. Neurol. Ther..

[6] Kavaliunas A., Danylaite Karrenbauer V., Hillert J. (2021). Socioeconomic Consequences of Multiple Sclerosis—A Systematic Literature Review. Acta Neurol. Scand..

[7] Hilt Pfleger C.C., Meulengracht Flachs E., Koch-Henriksen N. (2010). Social Consequences of Multiple Sclerosis (1): Early Pension and Temporary Unemployment—A Historical Prospective Cohort Study. Mult. Scler. J..

[8] Reich D.S., Lucchinetti C.F., Calabresi P.A. (2018). Multiple Sclerosis. N. Engl. J. Med..

[9] Aust J.G., Gays F., Mickiewicz K.M., Buchanan E., Brooks C.G. (2009). The Expression and Function of the NKRP1 Receptor Family in C57BL/6 Mice. J. Immunol..

[10] Rozbeský D., Ivanova L., Hernychová L., Grobárová V., Novák P., Černý J. (2015). Nkrp1 Family, from Lectins to Protein Interacting Molecules. Molecules.

[11] Mesci A., Ljutic B., Makrigiannis A.P., Carlyle J.R. (2006). NKR-P1 Biology: From Prototype to Missing Self. Immunol. Res..

[12] Bialoszewska A., Malejczyk J. (2018). Biological and Clinical Significance of Human NKRP1A/LLT1 Receptor/Ligand Interactions. Crit. Rev. Immunol..

[13] Balato A., Unutmaz D., Gaspari A.A. (2009). Natural Killer T Cells: An Unconventional T-Cell Subset with Diverse Effector and Regulatory Functions. J. Investig. Dermatol..

[14] Maggi L., Santarlasci V., Capone M., Peired A., Frosali F., Crome S.Q., Querci V., Fambrini M., Liotta F., Levings M.K. (2010). CD161 Is a Marker of All Human IL-17-Producing T-Cell Subsets and Is Induced by RORC. Eur. J. Immunol..

[15] Woodfin A., Voisin M.-B., Nourshargh S. (2007). PECAM-1: A Multi-Functional Molecule in Inflammation and Vascular Biology. Arterioscler. Thromb. Vasc. Biol..

[16] Muller W.A., Weigl S.A., Deng X., Phillips D.M. (1993). PECAM-1 Is Required for Transendothelial Migration of Leukocytes. J. Exp. Med..

[17] Solowiej A., Biswas P., Graesser D., Madri J.A. (2003). Lack of Platelet Endothelial Cell Adhesion Molecule-1 Attenuates Foreign Body Inflammation Because of Decreased Angiogenesis. Am. J. Pathol..

[18] Newman P.J., Newman D.K. (2003). Signal Transduction Pathways Mediated by PECAM-1. Arterioscler. Thromb. Vasc. Biol..

[19] Elovaara I., Ukkonen M., Leppäkynnäs M., Lehtimäki T., Luomala M., Peltola J., Dastidar P. (2000). Adhesion Molecules in Multiple Sclerosis: Relation to Subtypes of Disease and Methylprednisolone Therapy. Arch. Neurol..

[20] Rössler K., Neuchrist C., Kitz K., Scheiner O., Kraft D., Lassmann H. (1992). Expression of Leucocyte Adhesion Molecules at the Human Blood-Brain Barrier (BBB). J. Neurosci. Res..

[21] Cannella B., Raine C.S. (1995). The Adhesion Molecule and Cytokine Profile of Multiple Sclerosis Lesions. Ann. Neurol..

[22] Minagar A., Jy W., Jimenez J.J., Sheremata W.A., Mauro L.M., Mao W.W., Horstman L.L., Ahn Y.S. (2001). Elevated Plasma Endothelial Microparticles in Multiple Sclerosis. Neurology.

[23] Kuenz B., Lutterotti A., Khalil M., Ehling R., Gneiss C., Deisenhammer F., Reindl M., Berger T. (2005). Plasma Levels of Soluble Adhesion Molecules SPECAM-1, SP-Selectin and SE-Selectin Are Associated with Relapsing-Remitting Disease Course of Multiple Sclerosis. J. Neuroimmunol..

[24] Losy J., Niezgoda A., Wender M. (1999). Increased Serum Levels of Soluble PECAM-1 in Multiple Sclerosis Patients with Brain Gadolinium-Enhancing Lesions. J. Neuroimmunol..

[25] Wimmer I., Tietz S., Nishihara H., Deutsch U., Sallusto F., Gosselet F., Lyck R., Muller W.A., Lassmann H., Engelhardt B. (2019). PECAM-1 Stabilizes Blood-Brain Barrier Integrity and Favors Paracellular T-Cell Diapedesis Across the Blood-Brain Barrier During Neuroinflammation. Front. Immunol..

[26] Poggi A., Zocchi M.R., Costa P., Ferrero E., Borsellino G., Placido R., Galgani S., Salvetti M., Gasperini C., Ristori G. (1999). IL-12-Mediated NKRP1A Up-Regulation and Consequent Enhancement of Endothelial Transmigration of Vδ2+ TCRγδ+ T Lymphocytes from Healthy Donors and Multiple Sclerosis Patients. J. Immunol..

[27] Zarobkiewicz M.K., Wawryk-Gawda E., Kowalska W., Janiszewska M., Bojarska-Junak A. (2021). Γδ T Lymphocytes in Asthma: A Complicated Picture. Arch. Immunol. Ther. Exp..

[28] Poggi A., Zocchi M.R., Carosio R., Ferrero E., Angelini D.F., Galgani S., Caramia M.D., Bernardi G., Borsellino G., Battistini L. (2002). Transendothelial Migratory Pathways of V 1+TCR + and V 2+TCR + T Lymphocytes from Healthy Donors and Multiple Sclerosis Patients: Involvement of Phosphatidylinositol 3 Kinase and Calcium Calmodulin-Dependent Kinase II. J. Immunol..

[29] Zarobkiewicz M.K., Kowalska W., Halczuk P., Woś J., Jodłowska-Jędrych B., Rejdak K., Roliński J., Bojarska-Junak A.A. (2019). RORγT Is Overexpressed in INKT and Γδ T Cells during Relapse in Relapsing-Remitting Multiple Sclerosis. J. Neuroimmunol..

[30] Bekkema R., Tadema A., Daenen S.M.G.J., Kluin-Nelemans H.C., Mulder A.B. (2008). An Improved Flow Cytometric Method Using FACS Lysing Solution for Measurement of ZAP-70 Expression in B-Cell Chronic Lymphocytic Leukemia. Cytometry B Clin. Cytom..

[31] Joe E., Frey T. (2008). Performance Comparison of Commercial Fixing and Permeabilizing Reagents. Blood.

[32] Jason J., Inge K.L. (2000). Mitogen-Induced Modulation of CD3, CD4, and CD8. Hum. Immunol..

[33] Abuzakouk M., Kelleher D., Feighery C., O’Farrelly C. (1996). Increased HLA-DR and Decreased CD3 on Human Intestinal Intraepithelial Lymphocytes: Evidence of Activation?. Gut.

[34] Prado-Garcia H., Aguilar-Cazares D., Meneses-Flores M., Morales-Fuentes J., Lopez-Gonzalez J.S. (2008). Lung Carcinomas Do Not Induce T-Cell Apoptosis via the Fas/Fas Ligand Pathway but Down-Regulate CD3 Epsilon Expression. Cancer Immunol. Immunother..

[35] Valle A., Barbagiovanni G., Jofra T., Stabilini A., Perol L., Baeyens A., Anand S., Cagnard N., Gagliani N., Piaggio E. (2015). Heterogeneous CD3 Expression Levels in Differing T Cell Subsets Correlate with the In Vivo Anti-CD3–Mediated T Cell Modulation. J. Immunol..

[36] Ross E.A., Coughlan R.E., Flores-Langarica A., Bobat S., Marshall J.L., Hussain K., Charlesworth J., Abhyankar N., Hitchcock J., Gil C. (2011). Cd31 Is Required on Cd4+ T Cells to Promote T Cell Survival during Salmonella Infection. J. Immunol..

[37] Marelli-Berg F.M., Clement M., Mauro C., Caligiuri G. (2013). An Immunologist’s Guide to CD31 Function in T-Cells. J. Cell Sci..

[38] Ashman L.K., Aylett G.W. (1991). Expression of CD31 Epitopes on Human Lymphocytes: CD31 Monoclonal Antibodies Differentiate between Naive (CD45RA+) and Memory (CD45RA−) CD4-positive T Cells. Tissue Antigens.

[39] Poggi A., Zancolli M., Catellani S., Borsellino G., Battistini L., Zocchi M.R. (2007). Migratory Pathways of T Cells and Response to CXCR3 and CXCR4 Ligands: Adhesion Molecules Involved and Implications for Multiple Sclerosis Pathogenesis. Ann. N. Y. Acad. Sci..

[40] Schirmer L., Rothhammer V., Hemmer B., Korn T. (2013). Enriched CD161high CCR6+ Γδ T Cells in the Cerebrospinal Fluid of Patients with Multiple Sclerosis. JAMA Neurol..

[41] Annibali V., Ristori G., Angelini D.F., Serafini B., Mechelli R., Cannoni S., Romano S., Paolillo A., Abderrahim H., Diamantini A. (2011). CD161highCD8+T Cells Bear Pathogenetic Potential in Multiple Sclerosis. Brain.

[42] Brundula V., Rewcastle N.B., Metz L.M., Bernard C.C., Yong V.W. (2002). Targeting Leukocyte MMPs and Transmigration: Minocycline as a Potential Therapy for Multiple Sclerosis. Brain.

[43] Schreiner T.G., Romanescu C., Popescu B.O. (2022). The Blood–Brain Barrier—A Key Player in Multiple Sclerosis Disease Mechanisms. Biomolecules.

[44] Rice G.P.A., Hartung H.-P., Calabresi P.A. (2005). Anti-A4 Integrin Therapy for Multiple Sclerosis: Mechanisms and Rationale. Neurology.

[45] Hawke S., Zinger A., Juillard P.-G., Holdaway K., Byrne S.N., Grau G.E. (2020). Selective Modulation of Trans-Endothelial Migration of Lymphocyte Subsets in Multiple Sclerosis Patients under Fingolimod Treatment. J. Neuroimmunol..

[46] Zarobkiewicz M.K., Kowalska W., Roliński J., Bojarska-Junak A.A. (2019). Γδ T Lymphocytes in the Pathogenesis of Multiple Sclerosis and Experimental Autoimmune Encephalomyelitis. J. Neuroimmunol..

[47] Bühler U., Fleischer V., Luessi F., Rezk A., Belikan P., Graetz C., Gollan R., Wolf C., Lutz J., Bar-Or A. (2017). Role of IL-17-Producing Lymphocytes in Severity of Multiple Sclerosis upon Natalizumab Treatment. Mult. Scler. J..

