# Supplementary material for: PECAM-1 Is Down-Regulated in γδT Cells during Remission, but Up-Regulated in Relapse of Multiple Sclerosis
Source: J Clin Med. 2022 Jun 4;11(11):3210. doi: 10.3390/jcm11113210 (PMC9181399; doi:10.3390/jcm11113210)
Supplement: Supplementary file 1 [file jcm-11-03210-s001.zip › Supplementary Table S1.pdf]

Supplementary Table S1. The detailed configuration of cytometers

BD FACS Canto II			
No	Laser	Filter setup	Detector name
1	405nm	450/50	Pacific Blue
2		510/50	AmCyan
3	488nm	530/30	FITC
4		585/42	PE
5		670LP mirror	PerCp-Cy5.5
6		780/60	PE-Cy7
7	633nm	660/20	APC
8		780/60	APC-Cy7
BD FACS Aria IIu			
		etup	r name
1	488nm	530/30	FITC
2		576/26	PE
3		610/20	PE-TexasRed
4		695/40	PerCp-Cy5.5
5		780/60	PE-Cy7
6	633nm	660/20	APC
7		780/60	APC-Cy7
Beckman Coulter Cytoflex LX			
No	Laser	Filter setup	Detector name

1	375nm	450/45	NUV450
2		525/40	NUV525
3		675/30	NUV675
4	405nm	450/45	Pacific Blue
5		525/40	Krome Orange
6		610/20	V610
7		660/10	V660
8		763/43	V763
9	488nm	525/40	FITC
10		610/20	ECD
11		690/50	PE-Cy5.5
12	561nm	585/42	PE
13		610/20	mCherry
14		675/30	PE-Cy5
15		710/50	PE-Cy5.5
16		763/43	PE-Cy7
17	638nm	660/10	APC
18		712/25	APC-A700
19		763/43	APC-A750
20	808nm	840/20	Alexa Fluor 790
21		885/40	IR885
