# Supplementary material for: PECAM-1 Is Down-Regulated in γδT Cells during Remission, but Up-Regulated in Relapse of Multiple Sclerosis
Source: J Clin Med. 2022 Jun 4;11(11):3210. doi: 10.3390/jcm11113210 (PMC9181399; doi:10.3390/jcm11113210)
Supplement: Supplementary file 1 [file jcm-11-03210-s001.zip › Supplementary Table S2.pdf]

Table S2A **Spearman correlation matrix for major clinical and immunological parameters, rho values are presented. ARR – annual relapse rate; EDSS – expanded disability status score; cd3, cd31 and cd161 were MFI (mean fluorescent intensity) values.**
*Values written in **bold** have p<0.05; detailed p values are presented in Fig. S2B*

	Age	Smoking	EDSS	ARR	years from diagnosis	d1	d1 cd3 mfi	d1 cd31	d1 cd161	d2	d2 cd3 mfi	d2 cd31	d2 cd161	gd	gd cd3 mfi	gd cd31	gd cd161	dim	bright
Age	1,00	-0,24	0,20	-0,12	0,47	-0,13	0,19	-0,34	0,05	-0,28	0,18	-0,14	0,02	-0,17	0,16	-0,12	0,01	**-0,52**	**0,53**
Smoking	-0,24	1,00	**-0,53**	0,51	**-0,54**	0,00	-0,41	0,49	0,04	-0,15	-0,48	0,48	0,00	-0,04	**-0,53**	0,37	-0,14	0,08	-0,04
EDSS	0,20	**-0,53**	1,00	0,23	0,49	0,38	-0,27	-0,16	-0,11	0,43	-0,05	-0,21	-0,28	**0,51**	-0,12	-0,26	-0,23	-0,17	0,15
ARR	-0,12	0,51	0,23	1,00	-0,28	0,42	**-0,65**	**0,61**	-0,14	0,36	**-0,49**	0,37	-0,24	0,42	**-0,53**	0,33	-0,22	0,37	-0,32
years from diagnosis	0,47	**-0,54**	0,49	-0,28	1,00	-0,05	0,22	-0,29	-0,06	0,15	0,32	-0,15	-0,13	0,22	0,42	-0,20	-0,05	-0,34	0,36
d1	-0,13	0,00	0,38	0,42	-0,05	1,00	-0,34	-0,01	-0,27	0,46	**-0,51**	0,08	-0,31	**0,70**	-0,42	-0,04	-0,26	0,20	-0,19
d1 cd3 mfi	0,19	-0,41	-0,27	**-0,65**	0,22	-0,34	1,00	**-0,68**	0,29	-0,33	**0,75**	**-0,54**	0,46	-0,44	**0,77**	**-0,52**	0,39	-0,03	0,00
d1 cd31	-0,34	0,49	-0,16	**0,61**	-0,29	-0,01	**-0,68**	1,00	-0,28	0,29	**-0,54**	0,46	-0,33	0,24	-0,48	**0,55**	-0,20	0,40	-0,34
d1 cd161	0,05	0,04	-0,11	-0,14	-0,06	-0,27	0,29	-0,28	1,00	-0,11	0,49	-0,49	**0,93**	-0,27	0,38	**-0,57**	**0,83**	0,11	-0,19
d2	-0,28	-0,15	0,43	0,36	0,15	0,46	-0,33	0,29	-0,11	1,00	-0,30	-0,03	-0,29	**0,80**	-0,24	-0,24	-0,13	0,15	-0,16
d2 cd3 mfi	0,18	-0,48	-0,05	**-0,49**	0,32	**-0,51**	**0,75**	**-0,54**	0,49	-0,30	1,00	**-0,69**	**0,65**	**-0,57**	**0,91**	-0,50	**0,55**	0,09	-0,12
d2 cd31	-0,14	0,48	-0,21	0,37	-0,15	0,08	**-0,54**	0,46	-0,49	-0,03	**-0,69**	1,00	**-0,55**	0,19	**-0,55**	**0,83**	-0,36	0,01	0,06
d2 cd161	0,02	0,00	-0,28	-0,24	-0,13	-0,31	0,46	-0,33	**0,93**	-0,29	**0,65**	**-0,55**	1,00	-0,43	**0,54**	-0,49	**0,90**	0,25	-0,31
gd	-0,17	-0,04	**0,51**	0,42	0,22	**0,70**	-0,44	0,24	-0,27	**0,80**	**-0,57**	0,19	-0,43	1,00	-0,42	-0,16	-0,31	0,15	-0,13
gd cd3 mfi	0,16	**-0,53**	-0,12	**-0,53**	0,42	-0,42	**0,77**	-0,48	0,38	-0,24	**0,91**	**-0,55**	**0,54**	-0,42	1,00	-0,41	**0,58**	0,12	-0,16
gd cd31	-0,12	0,37	-0,26	0,33	-0,20	-0,04	**-0,52**	**0,55**	**-0,57**	-0,24	-0,50	**0,83**	-0,49	-0,16	-0,41	1,00	-0,31	0,14	-0,06
gd cd161	0,01	-0,14	-0,23	-0,22	-0,05	-0,26	0,39	-0,20	**0,83**	-0,13	0,55	-0,36	**0,90**	-0,31	**0,58**	-0,31	1,00	0,33	-0,39
dim	**-0,52**	0,08	-0,17	0,37	-0,34	0,20	-0,03	0,40	0,11	0,15	0,09	0,01	0,25	0,15	0,12	0,14	0,33	1,00	**-0,99**
bright	**0,53**	-0,04	0,15	-0,32	0,36	-0,19	0,00	-0,34	-0,19	-0,16	-0,12	0,06	-0,31	-0,13	-0,16	-0,06	-0,39	**-0,99**	1,00

Table S2B Detailed *p* values for correlation matrix. Where p<0.05, value is marked with green colour

	Age	Smoking	EDSS	ARR	years from diagnosis	d1	d1 cd3 mfi	d1 cd31	d1 cd161	d2	d2 cd3 mfi	d2 cd31	d2 cd161	gd	gd cd3 mfi	gd cd31	gd cd161	dim	bright
Age	.	0,404	0,444	0,643	0,065	0,607	0,458	0,190	0,845	0,268	0,482	0,599	0,945	0,509	0,561	0,650	0,987	0,042	0,038
Smoking	0,404	.	0,022	0,066	0,033	1,000	0,132	0,067	0,933	0,618	0,059	0,059	1,000	0,941	0,033	0,200	0,686	0,817	0,933
EDSS	0,444	0,022	.	0,376	0,056	0,137	0,286	0,547	0,696	0,088	0,835	0,413	0,277	0,039	0,667	0,329	0,412	0,517	0,577
ARR	0,643	0,066	0,376	.	0,299	0,093	0,006	0,014	0,593	0,150	0,049	0,145	0,351	0,092	0,037	0,204	0,427	0,160	0,225
years from diagnosis	0,065	0,033	0,056	0,299	.	0,856	0,402	0,298	0,828	0,578	0,225	0,581	0,640	0,405	0,120	0,464	0,857	0,210	0,184
d1	0,607	1,000	0,137	0,093	0,856	.	0,183	0,972	0,313	0,067	0,037	0,753	0,230	0,002	0,107	0,897	0,338	0,465	0,489
d1 cd3 mfi	0,458	0,132	0,286	0,006	0,402	0,183	.	0,005	0,278	0,201	0,001	0,026	0,065	0,082	0,001	0,040	0,149	0,926	1,000
d1 cd31	0,190	0,067	0,547	0,014	0,298	0,972	0,005	.	0,288	0,283	0,032	0,074	0,208	0,361	0,062	0,030	0,482	0,126	0,192
d1 cd161	0,845	0,933	0,696	0,593	0,828	0,313	0,278	0,288	.	0,689	0,059	0,056	0,000	0,315	0,148	0,024	0,000	0,681	0,491
d2	0,268	0,618	0,088	0,150	0,578	0,067	0,201	0,283	0,689	.	0,235	0,913	0,255	0,000	0,367	0,367	0,648	0,571	0,549
d2 cd3 mfi	0,482	0,059	0,835	0,049	0,225	0,037	0,001	0,032	0,059	0,235	.	0,003	0,005	0,018	0,000	0,052	0,035	0,755	0,649
d2 cd31	0,599	0,059	0,413	0,145	0,581	0,753	0,026	0,074	0,056	0,913	0,003	.	0,023	0,455	0,031	0,000	0,182	0,969	0,822
d2 cd161	0,945	1,000	0,277	0,351	0,640	0,230	0,065	0,208	0,000	0,255	0,005	0,023	.	0,085	0,034	0,057	0,000	0,355	0,244
gd	0,509	0,941	0,039	0,092	0,405	0,002	0,082	0,361	0,315	0,000	0,018	0,455	0,085	.	0,109	0,563	0,265	0,586	0,625
gd cd3 mfi	0,561	0,033	0,667	0,037	0,120	0,107	0,001	0,062	0,148	0,367	0,000	0,031	0,034	0,109	.	0,112	0,026	0,649	0,563
gd cd31	0,650	0,200	0,329	0,204	0,464	0,897	0,040	0,030	0,024	0,367	0,052	0,000	0,057	0,563	0,112	.	0,259	0,617	0,814
gd cd161	0,987	0,686	0,412	0,427	0,857	0,338	0,149	0,482	0,000	0,648	0,035	0,182	0,000	0,265	0,026	0,259	.	0,237	0,153
dim	0,042	0,817	0,517	0,160	0,210	0,465	0,926	0,126	0,681	0,571	0,755	0,969	0,355	0,586	0,649	0,617	0,237	.	0,000
bright	0,038	0,933	0,577	0,225	0,184	0,489	1,000	0,192	0,491	0,549	0,649	0,822	0,244	0,625	0,563	0,814	0,153	0,000	.
